# Supplementary material for: Size-dependent deformation behavior in nanosized amorphous metals suggesting transition from collective to individual atomic transport
Source: Nat Commun. 2023 Sep 26;14:5987. doi: 10.1038/s41467-023-41582-2 (PMC10522620; doi:10.1038/s41467-023-41582-2)
Supplement: Supplementary file 1 — Supplementary Information [file 41467_2023_41582_MOESM1_ESM.pdf]

Supplementary Information for

Size-dependent Deformation Behavior in Nanosized Amorphous Metals Suggesting Transition from Collective to Individual Atomic Transport

Naijia Liu, Sungwoo Sohn, Min Young Na, Gi Hoon Park, Arindam Raj, Guannan Liu, Sebastian A. Kube, Fusen Yuan, Yanhui Liu, Hye Jung Chang, and Jan Schroers

*Corresponding author. Email: sungwoo.sohn@yale.edu, jan.schroers@yale.edu

This PDF file include:

Supplementary Figures 1 ~ 10

Supplementary Notes:

Supplementary Note 1. Model of viscous flow

Supplementary Note 2. Mechanism deviation from viscous flow

Supplementary Note 3. Model of diffusion

Supplementary Note 4. Composition and atomic structure characterization

Supplementary Note 5. Size and temperature dependency of mechanisms

Supplementary Note 6. Viscosity and diffusivity measurement

Supplementary Note 7. Deformation mechanism map

Supplementary Note 8. Breakdown of Stokes-Einstein relation

Supplementary Note 9. Bulk and interface glass transition

Supplementary References 1 ~ 10

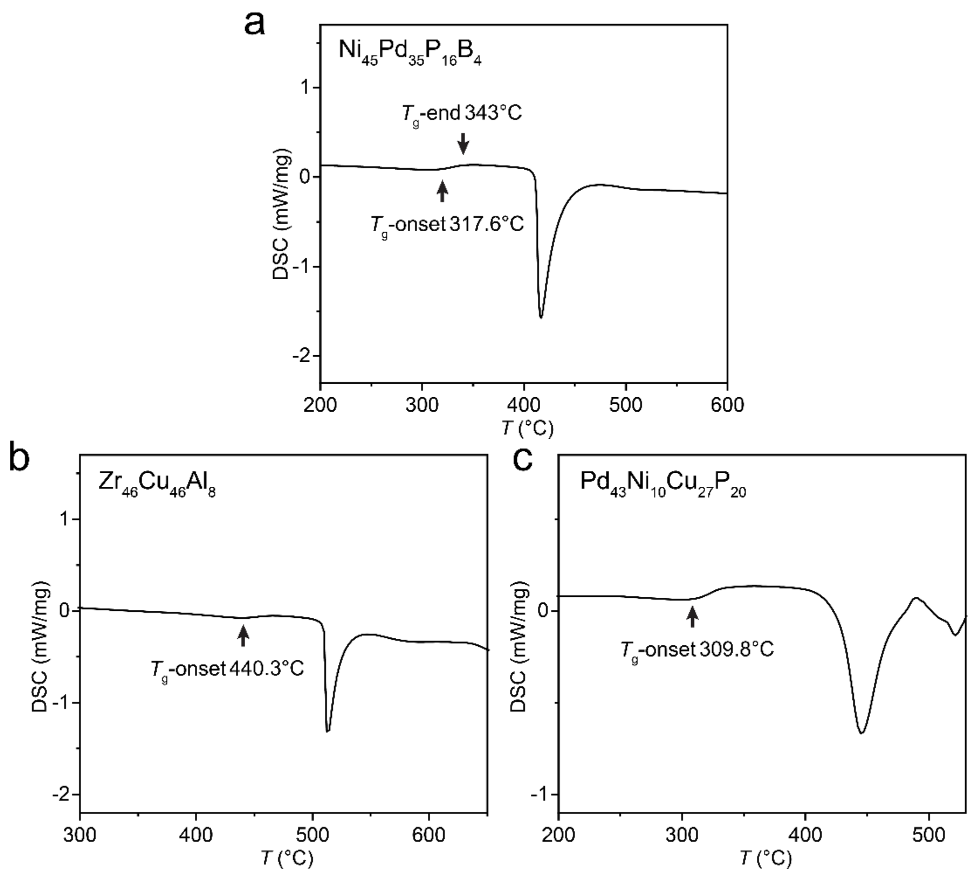

Supplementary Figure 1. DSC of Ni-based, Ze-based, and Pd-based BMGs which are used in this study as feedstock materials. a With a heating rate of 0.33°C/s, the calorimetric glass transition temperature for Ni-BMG is measured to be $T_g \sim 317$°C. The temperature range of onset to end of the glass transition is $\Delta T = 25.4$°C. **b** With a heating rate of 0.33°C/s, the calorimetric glass transition temperature for Zr-BMG is measured to be $T_g \sim 440$°C. **c** With a heating rate of 0.33°C/s, the calorimetric glass transition temperature for Pd-BMG is measured to be $T_g \sim 310$°C.

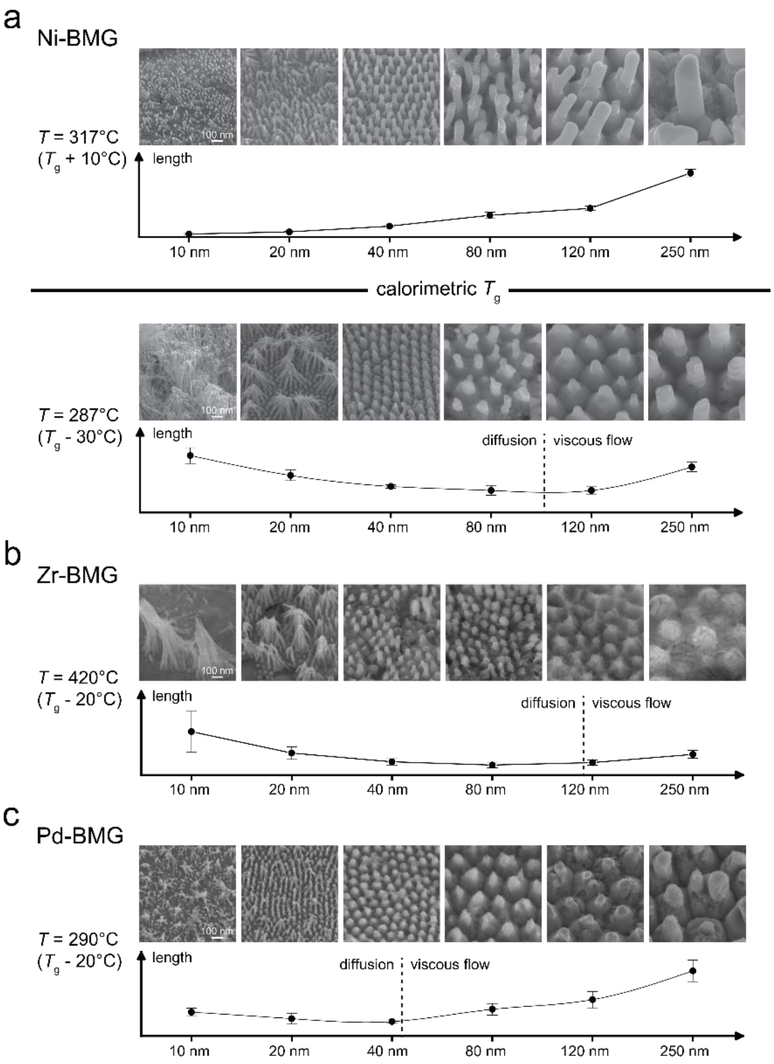

Supplementary Figure 2. Typical scaling behaviors of nanomolding. a When molded at 317°C (T_g + 10°C), the length of Ni-BMG nanowires increases with the molding diameter. When molded at 287°C (T_g – 30°C) the length of Ni-BMG nanowires first decreases and then increases with the molding diameter, with a transition size between 80 nm and 120 nm. The molding condition for 317°C is p = 1 GPa and t = 300 s, and for 287°C is p = 2 GPa and t = 36000 s. **b** Similar scaling behavior for Zr-BMG when molded at 420°C (T_g – 20°C). The length of Zr-BMG nanowires first decreases and then increases with the molding diameter, with a transition size between 80 nm and 120 nm. The molding condition is p = 1.25 GPa and t = 900 s. **c** Similar scaling behavior for Zr-BMG when molded at 290°C (T_g – 20°C). The length of Pd-BMG nanowires first decreases and then increases with the molding diameter, with a transition size around 40 nm. The molding condition is p = 2 GPa and t = 3600 s. Error bars are defined by the standard deviation of more than 10 measurements.

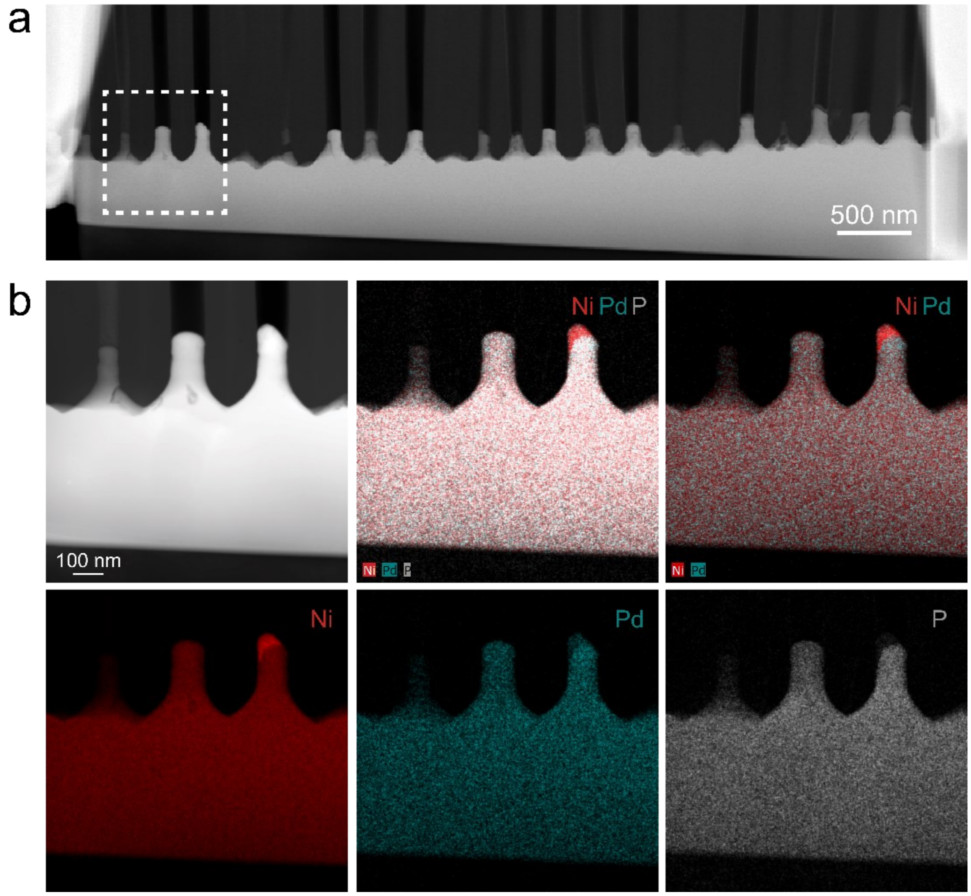

Supplementary Figure 3. Composition distribution of nanowires formed through collective atomic transport. a. STEM overview of 120 nm nanowires formed at 297°C. **b.** HAADF image and EDS mappings of Ni, Pd, and P of the location marked by dashed box in (a).

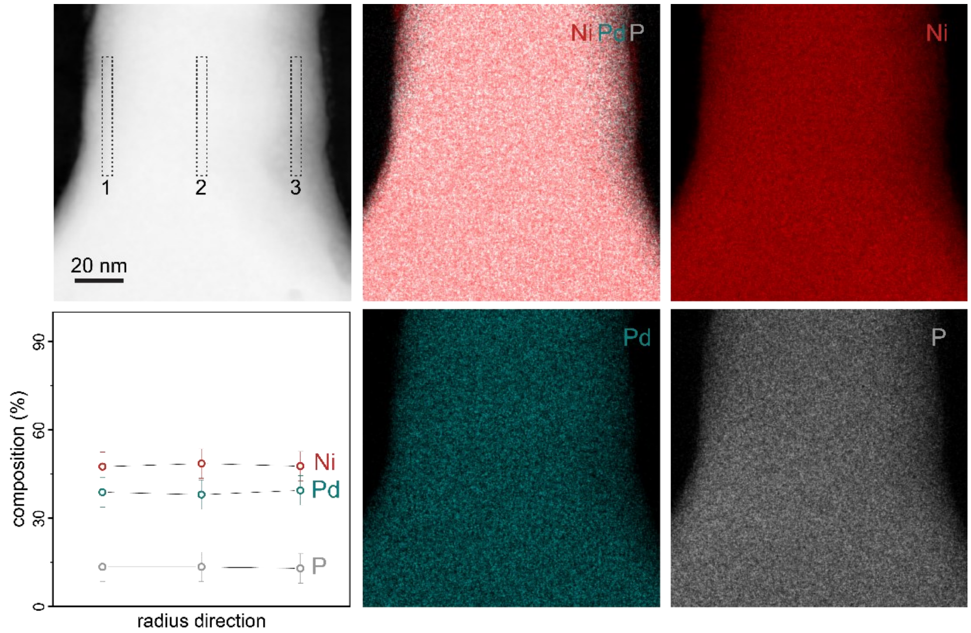

Supplementary Figure 4. Uniformity of the composition distribution along the radius direction in nanowires formed through collective atomic transport. STEM reveals a uniform distribution of the composition of all components from the center to the surface of the nanowire. Example shown is a 120 nm nanowire formed at 297°C (collective atomic transport). Error bars represent the measurement error of EDS.

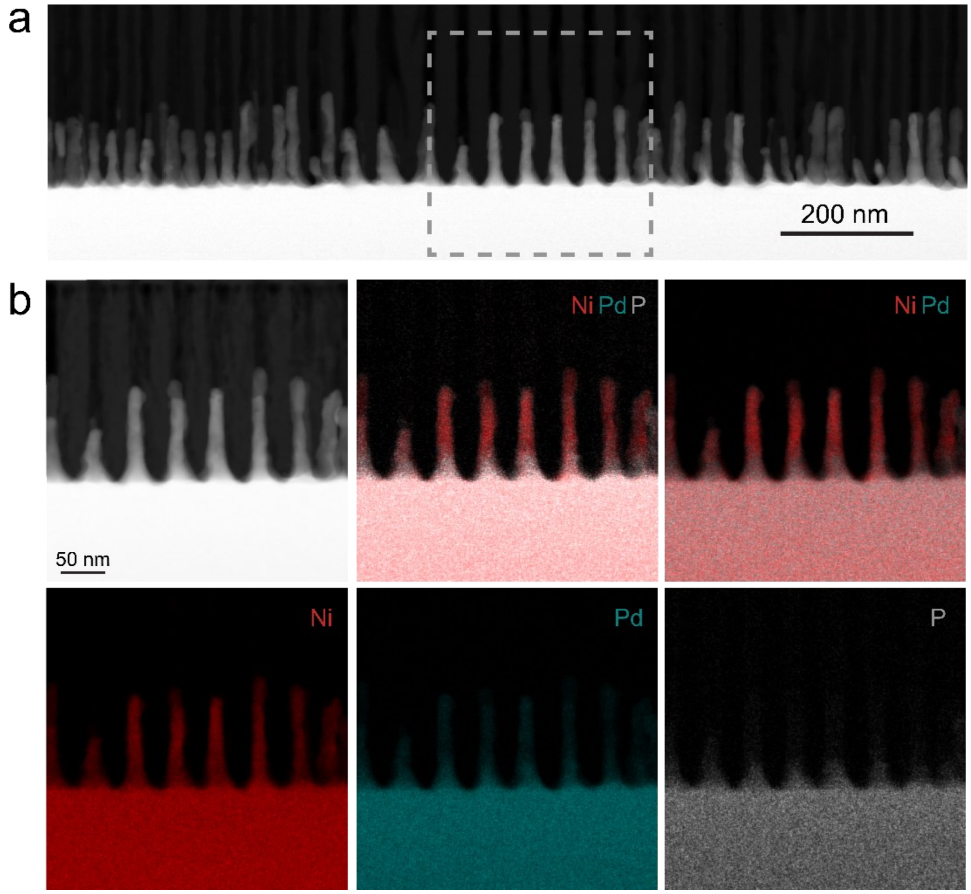

Supplementary Figure 5. Composition distribution of nanowires formed through individual atomic transport. a. STEM overview of 20 nm nanowires formed at 297°C. **b.** HAADF image and EDS mappings of Ni, Pd, and P of the location marked by dashed box in (a).

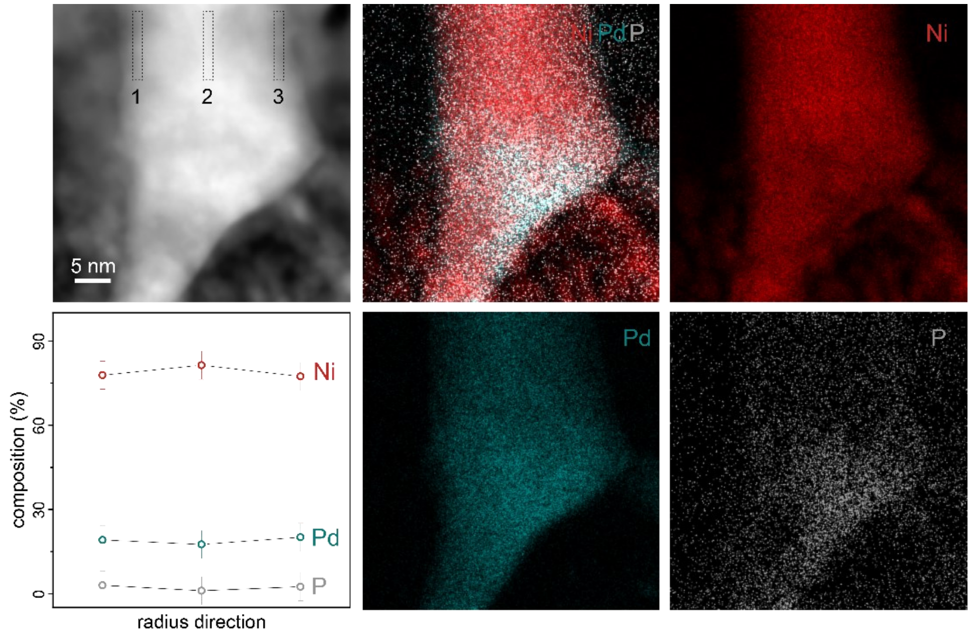

Supplementary Figure 6. Uniformity of the composition distribution along the radius direction in nanowires formed through individual atomic transport. STEM reveals a uniform distribution of the composition of all components from the center to the surface of the nanowire. Example shown is a 20 nm nanowire formed at 297°C (individual atomic transport). Error bars represent the measurement error of EDS.

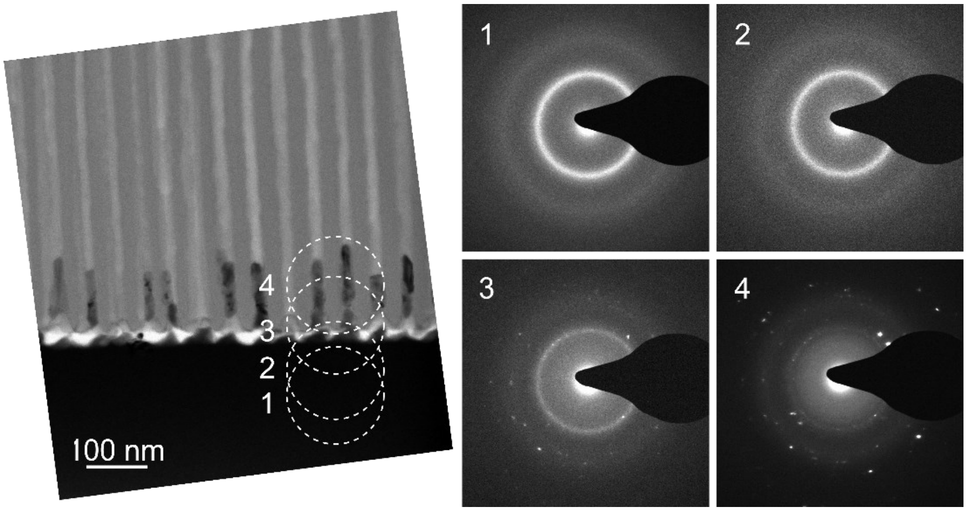

Supplementary Figure 7. Atomic structure at different locations of nanowires formed through individual atomic transport. Diffraction patterns from the substrate (1), substrate and root (2), substrate and nanowires (3), and nanowires (4) reveal a gradual change from completely amorphous to predominantly crystalline.

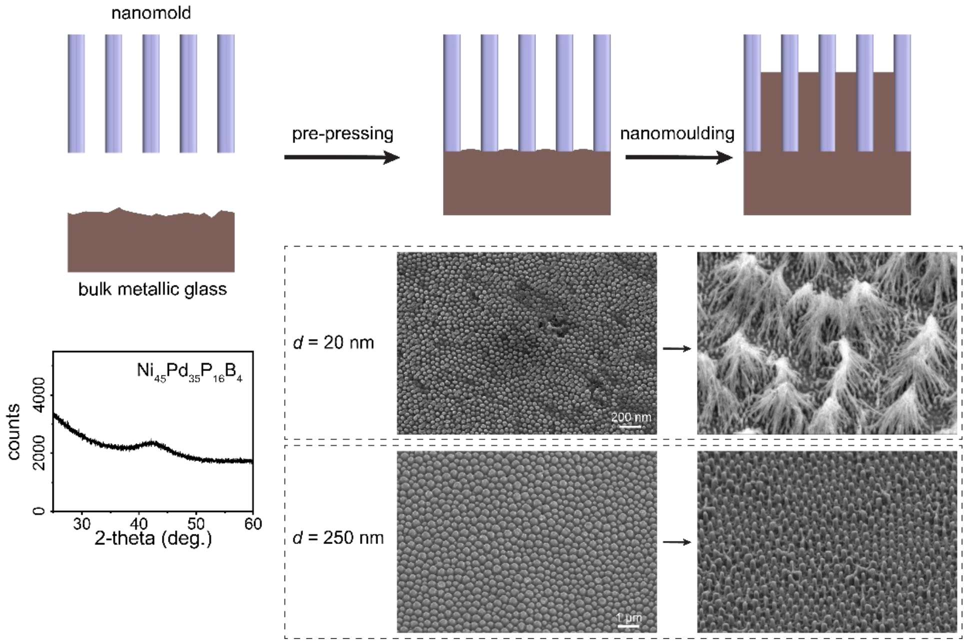

Supplementary Figure 8. Process of nanomolding. Amorphous feedstock material (Ni-based BMG, $Ni_{45}Pd_{35}P_{16}B_4$) is first pre-pressed against a hard mold with pre-defined nanopatterns to remove the surface roughness. Subsequently, nanomolding is carried out, which replicates the nano-mold, thereby creating nanowire arrays. Nanomolding is controlled through temperature, pressure, mold diameter, and molding time (T, p, d, and t). The condition for pre-pressing is fixed to T = 337°C (20°C above calorimetric T_g) with the pressure linearly increasesing from 0 to 3.5 MPa in 30 s and hold for 3 s, which has been confirmed able to sufficiently remove the surface roughness of the feedstock and result in only small prints with aspect ratio smaller than 0.5. Examples shown are SEM images of samples with d = 20 nm and 250 nm after pre-pressing and nanomolding (T = 297°C, p = 1.25 GPa, and t = 36000 s).

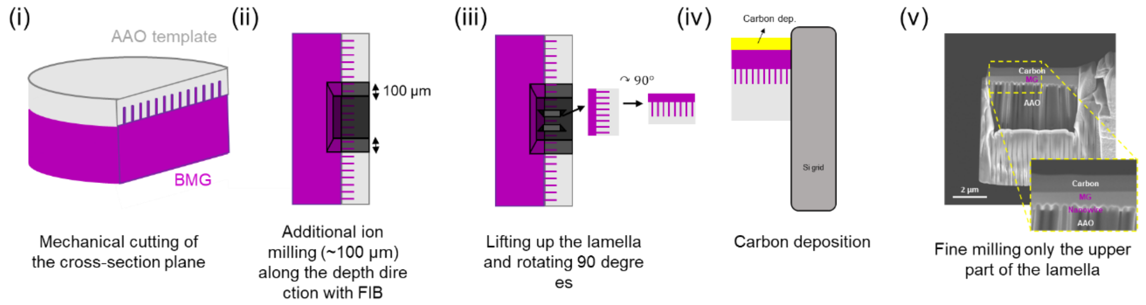

Supplementary Figure 9. Process of FIB sampling. The thin lamella samples for TEM used in this study were carefully prepared through focused ion beam, keeping the original structure.

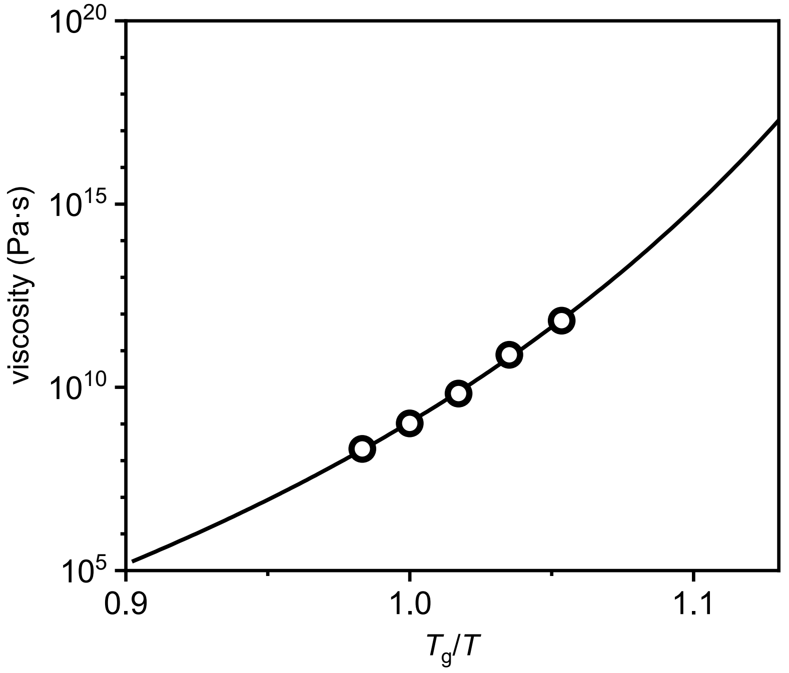

Supplementary Figure 10. Viscosity fitted with VFT equation using the experimentally determined nanowire length of 250 nm diameter. We use the results from the largest molding diameter, 250 nm, to estimate the viscosity of the Ni-based BMG for Fig. 1b. Viscosity of each molding temperature is obtained using eq. 1, and then used to fit the viscosity of the entire temperature range with the VFT equation. The fitting results reveal a fragility D^*_{VFT} = 17.8 and T_0 = 395 K, which are similar to reported data (D^*_{VFT} = 15.4, T_0 = 396 K for $Ni_{40}Pd_{40}P_{20}$ [1]). Details are discussed in Supplementary Note 2.

Supplementary Notes

Supplementary Note 1. Model of viscous flow

The deformation of amorphous materials is widely understood by viscous flow. In nanomolding, where the deformation of materials is confined in nanocavities with cylinder geometry, the deformation is in lamina flow. For a cylinder-shape cavity, Hagen–Poiseuille equation (eq. 1) describes the lamina flow behavior as:

$$p = \frac{32\eta}{t}(\frac{L}{d})^2 - \frac{4\gamma\cos\theta}{d}$$

, where *p*, *t*, and *d* are the molding conditions of pressure, time and diameter, *L* is the deformation length of the material, *η*, *γ*, and *θ* are the viscosity, surface tension, and the conducting angle of the material-mold interface.

Eq. 1 gives the length of nanowires in nanomolding as a function of the molding conditions and material properties in:

$$L = d\sqrt{\left(p + \frac{4\gamma\cos\theta}{d}\right)t/32\eta} \quad (1.1)$$

This equation shows a positive correlation between the length of nanowires (*L*) and the molding diameter (*d*) with all other moving conditions fixed.

In the case of nanomolding of bulk metallic glasses in this study, where $p > 1$ GPa, *d* is no less than 10 nm, by assuming $\gamma = 1$ N/m and $\theta = 120°$ [2], we have

$$\frac{4\gamma\cos\theta}{d} < \frac{4 * 1\ \text{N/m} * 0.5}{10\ \text{nm}} = 2 \times 10^8\ \text{Pa} < 20\%\ p$$

This affects $L \propto \sqrt{\left(p + \frac{4\gamma\cos\theta}{d}\right)}$ within a range of no more than 10 %. As a result, for high-order approximation, for the molding conditions in the current study we can neglect the $\frac{4\gamma\cos\theta}{d}$ term and have:

$$L = d\sqrt{\frac{pt}{32\eta}} \propto d \quad (1.2)$$

And the normalized length, *L'*, is:

$$L' = \frac{L}{\sqrt{pt}} = \sqrt{\frac{1}{32\eta}}d \propto d \quad (1.3)$$

This equation reveals a proportional relation between the normalized length of nanowires (*L'*) and the molding diameter (*d*) with other molding conditions fixed.

Supplementary Note 2. Mechanism deviation from viscous flow

We can further write the eq. 1.3 in the following form:

$$\ln(L') = \ln\left(\frac{L}{\sqrt{pt}}\right) = \ln\left(\sqrt{\frac{1}{32}}\right) - \ln\left(\frac{\sqrt{\eta}}{d}\right) \quad (2.1)$$

The equation above reveals a linear relationship between *L'* and $\eta^{1/2}/d$ when applying a log-plot as shown in Fig. 1b.

To achieve the viscosity data of the here-used Ni-based BMG and plot in Fig. 1b, we calculated with the length data (*L*) from nanomolding and apply Vogel–Fulcher–Tammann (VFT) fitting.

We used length data with a molding diameter (*d*) of 250 nm, which is the largest deformation length scale in this study, through the follow equation:

$$\eta = \left(p + \frac{4\gamma\cos\theta}{d}\right) t \cdot d^2 \frac{1}{32L^2} \quad (2.2)$$

We assumed the surface tension γ = 1 N/m and the contacting angle of nanowire-mold interface θ = 120°. By using data of 250 nm nanomolding processed at T_g + 10°C (327°C), T_g (317°C), T_g - 10°C (307°C), T_g - 20°C (297°C), and T_g - 30°C (287°C), we got the viscosity for each processing temperature (see details in Supplementary Fig. 10).

We further fitted the viscosity data from 250 nm molding with the Vogel–Fulcher–Tammann (VFT) equation:

$$\eta = \eta_0 \exp\left(\frac{D^*{}_{\mathrm{VFT}} T_0}{T - T_0}\right)$$

in the form of:

$$\ln\eta = \ln\eta_0 + \frac{D^*{}_{\mathrm{VFT}} T_0}{T - T_0} = \mathrm{A} + \frac{D^*{}_{\mathrm{VFT}} T_0}{T - T_0}$$

The fitted results (Supplementary Fig. 10) show fragility D^*_{VFT} = 17.8 and T_0 = 395 K, which both match with literature data (D^*_{VFT} = 15.4, T_0 = 396 K for $Ni_{40}Pd_{40}P_{20}$ [3]).

In Fig. 1b, length data from 30 nanomolding samples are presented. These samples are molded at five different temperatures, T_g + 10°C (327°C), T_g (317°C), T_g - 10°C (307°C), T_g - 20°C (297°C), and T_g - 30°C (287°C), and for each temperature with six different diameters, 10 nm, 20 nm, 40 nm, 80 nm, 120 nm, and 250 nm. For each sample, the normalized length $L' = \frac{L}{\sqrt{pt}}$ is calculated from experimental results and molding conditions and $\eta^{1/2}/d$ with the viscosities achieved from the VFT fitting using 250 nm molding data. The results show contrasting deformation behaviors among different molding temperatures. When the molding temperature is no less than the calorimetric glass transition temperature (T_g), T_g + 10°C (327°C) and T_g (317°C), the normalized length (*L'*) generally follows the straight line as predicted by eq. 2.1, showing a viscous flow mechanism. However, at lower molding temperatures, T_g - 10°C (307°C), T_g - 20°C (297°C), and T_g - 30°C

(287°C), the normalized length data (L') does not follow eq. 2.1 and shows a different deformation mechanism.

Supplementary Note 3. Model of diffusion

Atomic diffusion is another mechanism that can enable the deformation of amorphous materials in nanocavities. During nanomolding, the atomic diffusion process involves the transport of atoms driven by a gradient of chemical potential, which is realized in a pressure gradient, as described by the Fick's law:

$$J = \frac{D}{\mathrm{k_B}T}\nabla\mu = \frac{D}{\mathrm{k_B}T}\nabla(p\Omega) \tag{3.1}$$

, where J is the volume flux in the unit of m/s, μ is the chemical potential, D is the diffusivity, k_B is the Boltzmann constant, and Ω is the mean atomic volume in the feedstock material.

To quantify the deformation of nanowires in nanomolding, we consider the change rate of volume in the nanowire, $\mathrm{d}V/\mathrm{d}t$, resulted by the atomic diffusion volume flus (J) through a cross section area (S):

$$\frac{\mathrm{d}V}{\mathrm{d}t} = S \cdot J = \frac{SD}{\mathrm{k_B}T}\nabla(p\Omega) \tag{3..2}$$

In amorphous materials, the atomic transport along surface/interface is several orders of magnitude faster than that inside the bulk [1,4]. This makes the atomic diffusion through the interface a much more pronounced mechanism for deformation in nanomolding. We consider a diffusion process that happens along the interface layer between the nanowire and the mold, with $S = \pi d\delta$, where δ is the thickness of the interface layer (with a typical value of ~ 1 nm), and get:

$$\frac{\mathrm{d}L}{\mathrm{d}t} = \frac{\frac{\mathrm{d}V}{\mathrm{d}t}}{\frac{\pi d^2}{4}} = \frac{4\delta D_\mathrm{I} p\Omega}{dL\mathrm{k_B}T} \tag{3.3}$$

By integrate eq. 3.3 we have:

$$L = \sqrt{{L_0}^2 + \frac{8D_\mathrm{I} p\Omega t}{\mathrm{k_B}T}\frac{\delta}{d}} \tag{3.4}$$

Here L_0 is an integration constant that can be resulted by the loading process and the entry effect in nanomolding. In this study, as the loading time is controlled short compared to the molding time and error range in length from the entry effect is carefully removed through a pre-pressing process (Supplementary Fig. 8), eq. 3.4 comes to:

$$L = \sqrt{\frac{8D_\mathrm{I} p\Omega t}{\mathrm{k_B}T}\frac{\delta}{d}} \propto d^{-1/2} \tag{3.5}$$

When all other molding conditions are fixed, eq. 3.5 shows a decreasing scaling behavior of L with increasing molding diameter (d).

Supplementary Note 4. Composition and atomic structure characterization

To confirm the mechanism transition between individual and collective atomic transport and further study their effect on the chemistry and structure of the molded materials, we carried out FIB-TEM experiments. We chose the samples molded at T_g - 20°C (297°C) with d = 20 nm and 120 nm as the representative samples for individual and collective atomic transport respectively. Upon nanomolding, the combination of the substrate (bulk), the nanowires, and the mold was thinned down by focused-ion-beam for TEM characterization. This enables us to study the composition and structure change from the substrate to the nanowire.

We observed 41 nanowires with d = 120 nm (with 22 of them shown in Supplementary Fig. 3a), which are revealed to be through collective atomic transport via scaling experiments. These nanowires show uniform composition along the growth direction (Fig. 2a and Supplementary Fig. 3b) and radius direction (Supplementary Fig. 4), which is identical to the substrate (bulk) material, and maintains the amorphous structure from the feedstock (Fig. 3a and b). It is worthy to note that some of the 120 nm nanowires (~ 13 among 41 observed ones, with ~ 7 included in Supplementary Fig. 3a) have a very short "cap" at the tip (typical example as the right one shown in Supplementary Fig. 3b). These caps are with a non-round geometry, in contrast than the typical round tip formed through viscous flow (example as the middle one in Supplementary Fig. 3b). EDS measurements reveal these caps to be Ni enriched. And high-resolution TEM confirms that the caps have crystalline structures. These results provide another evidence of the competition between individual atomic transport (interface diffusion) and collective atomic transport (viscous flow), when viscous flow dominates the deformation of 120 nm wires at 297°C, diffusion can have secondary contribution and affect locally the composition and the atomic structure at the tip of formed nanowires.

We observed 101 nanowires with d = 20 nm (with 40 of them shown in Supplementary Fig. 5a), which are revealed to be through individual atomic transport via scaling experiments. These nanowires show gradually changing composition along the growth direction (Fig. 2b and Supplementary Fig. 5b) and uniform composition along the radius direction (Extended Data Fig. 6), from the substrate (bulk) material. As a result, their gradually change from amorphous to crystal along the growth direction (Fig. 3c to e). The amorphous-to-crystal transition in these nanowires typically happens at ~ 50 nm away from the entrance of the nanocavities (Fig. 3c to e), which is much longer than the length possibly formed through the pre-pressing process (L/d < 0.5).

It is also important to consider the potential effect through the interaction between the materials components and the inside wall of the nanomold. The interface interaction between the nanochannel and the nanowires is one important aspect that can potentially affect the atomic transport. The glass forming systems used in this study contain multiple elements. The possibility of chemical partitioning induced by different interface energies of these elements need to be considered. In principle, both interface diffusion mechanism and the chemical interaction between nanowires and nanochannels can induce a change in chemical composition. Whereas diffusion can only affect the composition in individual atomic transport case, the interface chemical interaction will affect both individual and collective atomic transport cases in an identical way. This is because the individual and collective atomic transport based nanomolding are performed at the same temperatures and the chemical interaction between Ni and Pd and Al_2O_3 will not change between

the two different deformation mechanisms. In our experimental result, however, a shift in chemical composition from the original BMG is only observed in the diffusion-dominated case, while the composition in viscous flow-dominated nanowires remains unchanged. This concludes the observed composition changes are due to un-equal diffusivities among elements in the nanochannels.

Supplementary Note 5. Size and temperature dependency of mechanisms

For each molding temperature, different molding diameters can be dominated by different mechanisms of individual (diffusion) and collective (viscous flow) atomic transport. As a significant criterion, we utilize the contrasting relation of L with d between the two mechanisms: L decreases with d in interface diffusion (eq. 3.5) vs. L increases with d in viscous flow (eq. 1.2) to define the underlying molding mechanism for each sample (as marked with blue and red symbols in Fig. 1d to f and Fig. 5). For example, when molded at 287°C (Supplementary Fig. 2), the length of nanowires decreases with diameter from $d = 10$ nm to $d = 80$ nm, and increases with diameter from $d = 120$ nm to $d = 250$ nm. This transition indicates the interface diffusion to dominate for d below 80 nm and viscous flow to dominate for d above 120 nm. As another example, when molded at 327°C (Extended Data Fig. 2), the length keeps increasing with d from $d = 10$ nm to $d = 250$ nm, indicating the viscous flow to be the dominating mechanism for all the samples.

In order to further characterize the critical diameter, d_c, we fit the length data with eq. 1.3 and eq. 3.5 and finds the cross-over of the two fitted curves which is associating with the diameter where the mechanism transition happens.

According to eq. 1.3, length data dominated by viscous flow is fitted with:

$$L' = \frac{L}{\sqrt{pt}} = \sqrt{\frac{1}{32\eta}}d = \mathrm{A_{visc}} \cdot d \tag{5.1}$$

, where $\mathrm{A_{visc}} = \sqrt{\frac{1}{32\eta}}$ is a constant to be fitted with the experimental data.

According to eq. 3.5, length data dominated by interface diffusion is fitted with

$$L' = \frac{L}{\sqrt{pt}} = \sqrt{\frac{8\Omega}{k_\mathrm{B}T} \cdot \delta D_\mathrm{I} \frac{1}{d}} = \sqrt{\frac{\mathrm{A_{diff}}}{d}} \tag{5.2}$$

, where $\mathrm{A_{diff}} = \frac{8\Omega}{k_B T}\delta D_\mathrm{I}$ is a constant to be fitted with the experimental data.

Supplementary Note 6. Viscosity and diffusivity measurement

Viscosity and interface diffusivity data at each molding temperature of the Ni-based BMG (shown as symbols in Fig. 6a) were obtained from the fitting results with eq. 5.1 and 5.2. Specifically, we calculated the viscosity η of each molding temperature with:

$$\eta = \frac{1}{32\mathrm{A_{visc}}^2} \tag{6.1}$$

, where A_{visc} is the constant fitted from eq. 5.1 ($A_{visc} = \sqrt{\frac{1}{32\eta}}$) using the length data in the viscous flow dominated regime. These results match well with the viscosity data of similar glass forming systems from literature [5]. As shown in Fig. 6a, the viscosity data with $T \geq 287°C$ follows a smooth curve, and the data for $T \leq 287°C$ follows a straight line with a dramatically different slope. A kink exists at $T = 287°C$.

We calculated the interface diffusivity D_I of each molding temperature with:

$$D_I = \frac{A_{diff} k_B T}{8\delta\Omega} \quad (6.2)$$

, where A_{diff} is the constant fitted from eq. 5.2 ($A_{diff} = \frac{8\Omega}{k_B T}\delta D_I$) using the length data in the diffusion dominated regime, and we assume δ to be 1 nm for calculation.

The above measured viscosity and interface diffusivity data of a series of molding temperatures (from T_g - 50°C to T_g + 10°C with an increment of 10°C between two molding temperatures) enable us to further fit η and D_I of the entire temperature range. Specifically, for viscosity, we used Vogel–Fulcher–Tammann (VFT) equation to fit the viscosity as a function of temperature in the equilibrium liquid state:

$$\eta = \eta_0 \exp\left(\frac{D^*_{VFT} T_0}{T - T_0}\right) \quad (6.3)$$

in the form of:

$$\ln \eta = \ln \eta_0 + \frac{D^*_{VFT} T_0}{T - T_0} = A + \frac{D^*_{VFT} T_0}{T - T_0}$$

, where A is a constant to be fitted using the measured viscosity data with $T \geq 287°C$. The fitting result reveals a VFT fragility of $D^*_{VFT} = 13.3$ and $T_0 = 414.7$ K, which both match well with reported data of similar glass forming systems in literatures ($D^*_{VFT} = 15.4$, $T_0 = 396$K for $Ni_{40}Pd_{40}P_{20}$[3]).

The kink in the measured viscosity at $T = 287°C$ indicates the bulk glass transition under the experimental conditions in nanomolding. Above $T = 287°C$, the molded material is in equilibrium liquid state with continuous structure reconfiguration when temperature shifts. Below $T = 287°C$, the molded material is in its glass state where a change in temperature does not result in structure reconfiguration and the temperature dependency of viscosity can be described by Arrhenius Law:

$$\eta = \eta_0' \exp\left(\frac{Q_\eta}{k_B T}\right) = \eta_0' \exp\left(\frac{Q_\eta}{k_B T_g} \cdot \frac{T_g}{T}\right) \quad (6.4)$$

, where Q_η is the activation energy of atomic motion through viscous flow. We fitted the viscosity data in the glass state by using a linear form of eq. 6.4:

$$\ln \eta = \ln \eta_0' + \frac{-Q_\eta}{k_B T_g} \cdot \frac{T_g}{T} = b + k \cdot \frac{T_g}{T}$$

, where b and k are constants to be fitted. The fitting result reveals k = 17.2 and $Q_\eta = 0.88$ eV.

We fitted the interface diffusivity data by Arrhenius Law:

$$D_{\mathrm{I}} = D_{\mathrm{I}\,0} \exp\left(\frac{-Q_{\mathrm{I}}}{k_{\mathrm{B}}T}\right) = D_{\mathrm{I}\,0} \exp\left(\frac{-Q_{\mathrm{I}}}{k_{\mathrm{B}}T_{\mathrm{g}}} \cdot \frac{T_{\mathrm{g}}}{T}\right) \qquad (6.5)$$

, where Q_{I} is the activation energy of interface diffusion. We fitted the interface diffusivity in the equilibrium liquid state with a linear form of eq. 6.5:

$$\ln D_{\mathrm{I}} = \ln D_{\mathrm{I}\,0} + \frac{-Q_{\mathrm{I}}}{k_{\mathrm{B}}T_{\mathrm{g}}} \cdot \frac{T_{\mathrm{g}}}{T} = \mathrm{b} + \mathrm{k} \cdot \frac{T_{\mathrm{g}}}{T}$$

, where b and k are constants to be fitted. The fitting results reveals k = - 31.1 and Q_{I} = 1.58 eV. This result matches well with the activation energy of surface diffusion for similar glass forming systems in literatures (for example, Q_{I} = 1.7 eV for Pt-based BMG [1]).

It is worth to note that the above viscosity and interface diffusivity data of each molding temperature, as calculated from the fitting results, are associated with an error range, e_{η} and e_{D} which prophages from the error range in the measurement of the normalized length, $e_{L'}$. In this study, as shown in Fig. 1d to f, Fig 5, and Supplementary Fig. 2, the error range of length and normalized length are defined by the standard deviation of more than 10 nanowires. We quantify the error range of the viscosity and interface diffusivity data through error propagation in the fitting process.

For viscosity, the results are fitted by eq. 5.1, $L' = \mathrm{A_{visc}} \cdot d$, where the constant $\mathrm{A_{visc}}$ is defined by least squares method:

$$\mathrm{A_{visc}} = \frac{\sum_{\mathrm{i=1}}^{\mathrm{n}} d_{\mathrm{i}} L'_{\mathrm{i}}}{\sum_{\mathrm{i=1}}^{\mathrm{n}} {d_{\mathrm{i}}}^2} \qquad (6.6)$$

, where n is the number of molding diameters among 10 nm, 20 nm, 40 nm, 80 nm, 120 nm, and 250 nm which are in the viscous flow dominated regime.

The error range of $\mathrm{A_{visc}}$ propagated from L' is:

$$e_{\mathrm{A\_visc}} = \sum_{\mathrm{i=1}}^{\mathrm{n}} \frac{\partial \mathrm{A_{visc}}}{\partial L'_{\mathrm{i}}} e_{L'} = \frac{\sum_{\mathrm{i=1}}^{\mathrm{n}} d_{\mathrm{i}} e_{L'_{\mathrm{i}}}}{\sum_{\mathrm{i=1}}^{\mathrm{n}} {d_{\mathrm{i}}}^2} \qquad (6.7)$$

And the error rage of η is:

$$e_{\eta} = \frac{\partial \eta}{\partial \mathrm{A_{visc}}} e_{\mathrm{A\_visc}} = \frac{2 e_{\mathrm{A\_visc}}}{32 {\mathrm{A_{visc}}}^3} \qquad (6.8)$$

For interface diffusivity, the results are fitted by eq. 5.2, $L' = \sqrt{\frac{\mathrm{A_{diff}}}{d}}$, where the constant $\mathrm{A_{diff}}$ is defined by least squares method:

$$\mathrm{A_{diff}} = \frac{\sum_{\mathrm{i=1}}^{\mathrm{n}} (1/d_{\mathrm{i}})(L'_{\mathrm{i}})^2}{\sum_{\mathrm{i=1}}^{\mathrm{n}} (1/{d_{\mathrm{i}}}^2)} \qquad (6.9)$$

the error range of $\mathrm{A_{diff}}$, propagated from L', is:

$$e_{\mathrm{A\_diff}} = \sum_{\mathrm{i=1}}^{\mathrm{n}} \frac{\partial \mathrm{A_{diff}}}{\partial (L'_{\mathrm{i}})^2} e_{(L'_{\mathrm{i}})^2} = \sum_{\mathrm{i=1}}^{\mathrm{n}} \frac{\partial \mathrm{A_{diff}}}{\partial (L'_{\mathrm{i}})^2} \cdot (2L'_{\mathrm{i}}) e_{L'_{\mathrm{i}}} = \frac{\sum_{\mathrm{i=1}}^{\mathrm{n}} (1/d_{\mathrm{i}}) \cdot (2L'_{\mathrm{i}}) e_{L'_{\mathrm{i}}}}{\sum_{\mathrm{i=1}}^{\mathrm{n}} (1/{d_{\mathrm{i}}}^2)} \qquad (6.10)$$

And:

$$e_{D_\mathrm{I}} = \frac{\partial D_\mathrm{I}}{\partial \mathrm{A}_\mathrm{diff}} e_{\mathrm{A\_diff}} = D_\mathrm{I} \frac{e_{\mathrm{A\_diff}}}{\mathrm{A}_\mathrm{diff}} \tag{6.11}$$

We applied the error analysis above to form the error bars with a logarithmic plot in Fig. 6a.

Supplementary Note 7. Deformation mechanism map

The deformation mechanism of amorphous materials is determined by size- and temperature-dependent, with the critical diameter of the mechanism transition, d_c, to be a function of the temperature.

The critical diameter, d_c, is defined where the two mechanisms of individual (interface diffusion) and collective (viscous flow) atomic transport give the same normalized growth length:

$$L' = \frac{L}{\sqrt{pt}} = \sqrt{\frac{8\Omega}{k_B T} * \delta D_\mathrm{I} \frac{1}{d}} = \sqrt{\frac{1}{32\eta}} d$$

This gives:

$$d_\mathrm{c} = (\frac{32 \times 8 \times \Omega}{k_\mathrm{B} T} \delta D_\mathrm{I} \eta)^{1/3} \tag{7.1}$$

, where both η and D_I are functions of the temperature.

By using the fitting results of eq. 6.3, 6.4, and 6.5, one can derive the expression of d_c to be:

$$\begin{cases} d_\mathrm{c} = (\frac{256\Omega}{k_\mathrm{B} T} \delta D_{\mathrm{I}\,0} \exp\left(\frac{-Q_\mathrm{I}}{k_\mathrm{B} T_\mathrm{g}} \cdot \frac{T_\mathrm{g}}{T}\right) \eta_0 \exp\left(\frac{D^*{}_\mathrm{VFT} T_0}{T - T_0}\right))^{1/3} & (T \geq 287°\mathrm{C}) \\ d_\mathrm{c} = (\frac{256\Omega}{k_\mathrm{B} T} \delta D_{\mathrm{I}\,0} \exp\left(\frac{-Q_\mathrm{I}}{k_\mathrm{B} T_\mathrm{g}} \cdot \frac{T_\mathrm{g}}{T}\right) \eta_0' \exp\left(\frac{Q_\eta}{k_\mathrm{B} T_\mathrm{g}} \cdot \frac{T_\mathrm{g}}{T}\right))^{1/3} & (T \leq 287°\mathrm{C}) \end{cases} \tag{7.2}$$

, where the fitting results of the parameter above are: $D_{\mathrm{I}\,0} = 7.729 \times 10^{-3}$ m^2/s, $Q_\mathrm{I} = 1.58$ eV; $\eta_0 = 2.34 \times 10^{-5}$ Pa·s, $D^*{}_\mathrm{VFT} = 13.3$, $T_0 = 414.7$ K; and $\eta_0' = 8.13 \times 10^3$ Pa·s, $Q_\eta = 0.88$ eV.

With eq. 7.2, the critical diameter achieves its maximum of 97 nm at 287°C, where the glass transition happens in this study. From eq. 7.1, the critical diameter is proportional to $(\eta D_\mathrm{I})^{1/3}$, which will increase with the ratio between η and $1/D_\mathrm{I}$ (and the gap between $\log_{10} \eta$ and $\log_{10} 1/D_\mathrm{I}$). In the temperature range above and below the glass transition (287°C in this study), as the viscosity is described by VFT equation and Arrhenius Law respectively, the gap between $\log_{10} \eta$ and $\log_{10} 1/D_\mathrm{I}$ increases and then decreases with decreasing temperature, and achieves its maximum at the glass transition. It is worthy to note that the temperature of max d_c can change when the experimental condition changes, but can be expected to be always at the condition-dependent glass transition temperature.

Supplementary Note 8. Breakdown of Stokes-Einstein relation

The data of viscosity and interface diffusivity revealed through nanomolding also provides us with a new tool to probe the breakdown of Stokes-Einstein relation in the supercooled liquid region. In the liquid phase, viscosity (η) and bulk diffusivity (D_B) are correlated through Stokes-Einstein equation:

$$D_B\eta = \frac{k_B T}{c\pi R} \tag{8.1}$$

, where R is the effective radius of the diffusing particle and c is a constant that varies between 2 to 3 according to the specific boundary conditions [6]. When temperature decreases and approaches the glass transition, the Stokes-Einstein relations breaks down and the change of bulk diffusivity and viscosity decouples.

With the data of nanomolding, the activation energy of interface diffusion is revealed to be Q_I = 1.58 eV (Supplementary Note 6). For bulk diffusivity, the random first-order transition (RFOT) theory predicts a free energy barrier for activated motion in the bulk to be double of that on the surface [4]. And thus, we set an activation energy for the bulk to be Q_B = 3.16 eV. And the bulk diffusivity is:

$$D_B = D_{B\,0}\exp\left(\frac{-Q_B}{k_B T}\right) = D_{B\,0}\exp\left(\frac{-Q_B}{k_B T_g}\cdot\frac{T_g}{T}\right) \tag{8.2}$$

And:

$$\ln D_B = \ln D_{B\,0} + \frac{-Q_B}{k_B T_g}\cdot\frac{T_g}{T}$$

, which gives:

$$\frac{\partial(\ln D_B)}{\partial(\frac{T_g}{T})} = \frac{-Q_B}{k_B T_g} \tag{8.3}$$

On the other hand, we can calculate the bulk diffusivity through the Stokes-Einstein relation, using the viscosity data, as:

$$\ln D_{SER} = \ln\left(\frac{k_B T_g}{c\pi R}/(\frac{T_g}{T})\right) + \ln\frac{1}{\eta} \tag{8.4}$$

Consider the fitting result from eq. 6.3, the above equation gives:

$$\frac{\partial(\ln D_{SER})}{\partial(\frac{T_g}{T})} = -\frac{1}{T_g/T} - \frac{\partial}{\partial\left(\frac{T_g}{T}\right)}\left(\frac{D^*{}_{VFT}\frac{T_g}{T}}{\frac{T_g}{T_0}-\frac{T_g}{T}}\right) = -\frac{1}{T_g/T} - \frac{D^*{}_{VFT}\frac{T_g}{T_0}}{(\frac{T_g}{T_0}-\frac{T_g}{T})^2} \tag{8.5}$$

At the breakdown point of Stokes-Einstein relation, the derivative of $\ln D_B$ and $\ln D_{SER}$ to T_g/T have the same value, resulting in a breakdown temperature T_{SER} to be:

$$\frac{-Q_{\mathrm{B}}}{k_{\mathrm{B}}T_{\mathrm{g}}} = -\frac{1}{T_{\mathrm{g}}/T_{\mathrm{SER}}} - \frac{D^{*}{}_{\mathrm{VFT}}\frac{T_{\mathrm{g}}}{T_0}}{\left(\frac{T_{\mathrm{g}}}{T_0} - \frac{T_{\mathrm{g}}}{T_{\mathrm{SER}}}\right)^2} \tag{8.6}$$

By considering the physical meaning of the solutions of eq. 8.6, we have $T_{\mathrm{g}}/T_{\mathrm{SER}}$ = 0.87626, and T_{SER} = 1.15 T_{g} ~ 408°C. This result also matches well with previous literatures where the breakdown temperature is measured to be ~ 1.2 T_{g} [7].

At T_{SER}, D_{B} and D_{SER} will give the same value of bulk diffusivity, this results in the expression of D_{B} to be (we set c = 3 and R = 125 pm in eq. 8.4):

$$D_{\mathrm{B}} = 8.70 \times 10^{7}\mathrm{m}^2/\mathrm{s} \cdot \exp\left(\frac{-3.16\ eV}{k_{\mathrm{B}}T}\right) \tag{8.7}$$

The calculated results are used for the plot in Fig. 6c.

Supplementary Note 9. Bulk and interface glass transition

The viscosity measurement with nanomolding (Supplementary Note 6) reveals a glass transition which occurs 30°C below the calorimetric T_{g}, while this transition behavior is not captured by the measurement of interface diffusivity. This result indicates a different glass transition in the bulk and at the interface.

In metallic glasses, the glass transition occurs when the relaxation time matches with the experimental time scale. Due to the fast atomic dynamics on the surface/interface, the relaxation time of surface/interface is much shorter than the bulk, and results in a lower glass transition temperature.

With the interface diffusivity (directly measured, eq. 6.5) and bulk diffusivity (indirectly measured, eq. 8.7) data, nanomolding offers the ability to probe atomic transport and relaxation both in the bulk and at the interface. We utilized this method to reveal the relaxation time and characterize the glass transition temperatures for both bulk and interface.

We obtained the relaxation time (τ) at the calorimetric T_{g} (τ_{g}) with DSC measurement (Supplementary Fig. 1) through [8]:

$$\tau_{\mathrm{g}} = \frac{\Delta T_{\mathrm{g}}}{a} \tag{9.1}$$

, where ΔT_{g} is the temperature window between T_{g}-onset (317.6°C) and T_{g}-end (343°C), and a is the DSC heating rate (0.33°C/s). By applying the DSC results, we measured the relaxation time to be τ_{g} = 76.2 s.

In metallic glasses, the bulk relaxation time (τ_{B}) is proportional to the viscosity (η), which follows the description of Vogel–Fulcher–Tammann (VFT) equation:

$$\tau_{\mathrm{B}} = \tau_{\mathrm{B}\,0} \exp\left(\frac{D^{*}{}_{\mathrm{VFT}}T_0}{T - T_0}\right) \tag{9.2}$$

, where D^*_{VFT} = 13.3 and T_0 = 414.7 K according to the fitting results in eq. 6.3. Considering τ_g = 76.2 s at calorimetric T_g, we have:

$$\tau_B = 1.67 \times 10^{-12}\ \text{s} \cdot \exp\left(\frac{13.3 \times 414.7\ \text{K}}{T - 414.7\ \text{K}}\right) \tag{9.3}$$

The relaxation time is different at the interface than in the bulk. This difference is due to the difference in the atomic kinetics, where the atomic mobility is much higher at the interface. Thus, the interface and bulk relaxation time (τ_I and τ_B) can be estimated to be proportional to the atomic mobility (diffusivity):

$$\tau_I = \tau_B \frac{D_I}{D_B} \tag{9.4}$$

With considering the results in eq. 6.5 and eq. 8.7, we have:

$$\tau_I = 1.50 \times 10^{-22}\ \text{s} \cdot \exp\left(\frac{13.3 \times 414.7\ \text{K}}{T - 414.7\ \text{K}}\right) \cdot \exp\left(\frac{1.58\ \text{eV}}{k_B T}\right) \tag{9.4}$$

τ_I and τ_B calculated by eq. 9.2 and eq. 9.4 are plotted in Fig. 6d.

Glass transition happens at the temperature where the relaxation time can match with the experimental time scale:

$$\tau \sim t_{\text{exp}} \tag{9.5}$$

In nanomolding with a controlled molding time to be t_{molding} = 36000 s, eq. 9.2 gives a bulk glass transition at temperature T = 288°C. This prediction is also proved by the viscosity kink at 287°C revealed by experiments. With the same experimental time scale of t_{molding} = 36000 s, eq. 9.4 gives an interface glass transition at temperature T = 254°C, which is 34°C lower than that of the bulk. The predicted temperature for interface glass transition is far below the range of molding temperature in this study as it requires much longer molding time or higher pressure than practical conditions to achieve enough length of nanowires for experimental measurements.

The calorimetric T_g is 29°C higher than the predicted temperature for bulk glass transition in this study. This is because of the shorter time scale in DSC measurement (~76.2 s) than in nanomolding. With t_{DSC} = 76.2 s, eq. 9.2 predicts the bulk T_g to be 317°C, and eq. 9.4 gives an interface T_g at 273°C. This result shows the interface glass transition temperature can be 44°C lower than the bulk glass transition temperature, which is reasonable according simulation results [9,10]. This gap between bulk and interface T_g, however, is relatively large compared to some experimental results [9]. This may be due to the interface diffusivity measurement in nanomolding, which is counting more contribution from the faster diffusors, can be larger than the average interface diffusivity in the material. This may result in a shorter estimated relaxation time at the interface and a lower interface glass transition temperature.

It is also worthy to note that the assumed value of Q_B = 3.16 eV in eq. 8.2 can also largely affect the predicted interface glass transition temperature. Although theory points $Q_B \sim 2Q_I$, an exact ratio of two between Q_B and Q_I is a rough estimation. For example, if Q_B is assumed to be 2.8 eV instead (a reasonable error range from Q_B = 3.16 eV of ~ 10%), the estimated breakdown temperature of Stokes-Einstein relation (eq. 8.6) is 437°C (~ 1.20 T_g), and the estimated interface glass transition temperature is 280°C (37°C lower than bulk T_g). If Q_B is assumed to be 2.5 eV instead (a reasonable

error range from Q_B = 3.16 eV of ~ 20%), the estimated breakdown temperature of Stokes-Einstein relation (eq. 8.6) is 470°C (~ 1.25 T_g), and the estimated interface glass transition temperature is 287°C (30°C lower than bulk T_g). These estimations may give a better matching with the reported experimental data with other methods [7,9].

Supplementary References

1 Zhang, P., Maldonis, J. J., Liu, Z., Schroers, J. & Voyles, P. M. Spatially heterogeneous dynamics in a metallic glass forming liquid imaged by electron correlation microscopy. *Nat. Commun.* **9**, 1-7 (2018).

2 Ding, S., Kong, J. & Schroers, J. Wetting of bulk metallic glass forming liquids on metals and ceramics. *J. Appl. Phys.* **110**, 043508 (2011).

3 Gallino, I., Schroers, J. & Busch, R. Kinetic and thermodynamic studies of the fragility of bulk metallic glass forming liquids. *J. Appl. Phys.* **108**, 063501 (2010).

4 Stevenson, J. D. & Wolynes, P. G. On the surface of glasses. *J. Chem. Phys.* **129**, 234514 (2008).

5 Kawamura, Y. & Inoue, A. Newtonian viscosity of supercooled liquid in a Pd 40 Ni 40 P 20 metallic glass. *Appl. Phys. Lett.* **77**, 1114-1116 (2000).

6 Hansen, J.-P. & McDonald, I. R. *Theory of simple liquids: with applications to soft matter*. (Academic press, 2013).

7 Bartsch, A., Rätzke, K., Meyer, A. & Faupel, F. Dynamic arrest in multicomponent glass-forming alloys. *Phys. Rev. Lett.* **104**, 195901 (2010).

8 Legg, B. A., Schroers, J. & Busch, R. Thermodynamics, kinetics, and crystallization of Pt57. 3Cu14. 6Ni5. 3P22. 8 bulk metallic glass. *Acta Mater.* **55**, 1109-1116 (2007).

9 Chatterjee, D. *et al.* Fast Surface Dynamics on a Metallic Glass Nanowire. *Acs Nano* **15**, 11309-11316 (2021).

10 Annamareddy, A., Voyles, P. M., Perepezko, J. & Morgan, D. Mechanisms of bulk and surface diffusion in metallic glasses determined from molecular dynamics simulations. *Acta Mater.* **209**, 116794 (2021).
